# Progress in Gynæcology

**Published:** 1895-02-16

**Authors:** 


### Progress in Gynæcology.

**Tamponning.**—Dr. Auvard,[1] of Paris, shows how much can be done for subacute and chronic salpingo-ovaritis by tamponning. The tampons are soaked in iodoform glycerine emulsion, and one is placed in the anterior, posterior, and lateral *culs de sac* each, and the cervix thus surrounded. The tampons are removed three times a week, and the treatment maintained for some weeks. None of the normal functions are interfered with, and the patient need not lay up entirely. The treatment is not only applicable to affections of the adnexa of the uterus, but also to the deviations of the organ itself, notably to displacements backwards.

**Ergot for Fibroids.**—Dr. Henri Leonard,[2] of Detroit, Mich., in a vigorous paper on "The Non-surgical Treatment of Fibroid Tumours of the Uterus," urges that the cardinal treatment to be adopted is the administration of ergot, first, last, and all the time. He administers it in full doses by the mouth, but combines sodium bromide with it, as he finds it steadies the action of the ergot, limits the back-ache symptom, and lessens the nausea induced by the continued use of ergot. He also applies ergot locally, per vaginam, and per rectum, and finds that it has a positive action on the pelvic blood vessels. He says: "My plan of treatment is anything and everything to limit the blood supply to the growing tumour, and so check its enlargement and stimulate the absorption of it when a retrograde metamorphosis takes place."

**Intra-Uterine Cauterisation.**—Dr. K. Sänger, of Leipzig, describes "An easy method of intra-uterine cauterisation," which is certainly worthy the attention of all who have to deal with cases of endometritis with ectropium of the cervix uteri, &c. As a matter of fact, the treatment by vaginal injections and remedial applications to the cervix are, in a great many cases, utterly useless. Most frequently, therefore, surgical operations, such as curetting, Schröeder's operation, &c., have to be resorted to, which, among other inconveniences, present the serious disadvantage of being frequently followed by relapse, especially in cases of catarrhal endometritis. He advocates the intermediate method of intra-uterine cauterisation. But the chief difficulty generally is that the cervix is not, as a rule, sufficiently open to allow the introduction of the caustic-holder; and ordinarily the sound can only be introduced after pulling down the uterus, which in these cases causes pain. It is easy to see, therefore, the advantage of a procedure which permits of practising intra-uterine cauterisation without pulling down the uterus, and without dilating the cervix. Dr. Sänger uses a sound of silver, twenty-two centimetres in length, fastened to a wooden handle eight centimetres long. The anterior third of this sound is flat, smooth, and a little more than a millimetre in breadth and half that in thickness. This sound, which is extremely flexible, bends easily, and keeps the form which is given it, adapts itself to all the sinuosities of the cervix, and can, therefore, be easily introduced even into the cervical canal of nulliparæ, and in cases where the uterus is abnormally anteflexed. A layer of cotton wool, varying in thickness according to the diameter of the cervical canal in each individual case, is twisted round the anterior third of the sound. Care should be taken that it be longer than the uterus, so that when the instrument is introduced far enough for the point to reach the fundus, a portion of the cotton layer is outside the external os. This charged sound is dipped into the solution used, and a speculum having been adjusted, the sound is introduced into the uterus as far as possible, and the end pushed up against the fundus. To remove the sound, it is grasped by forceps at a point covered by the protruding cotton, and by this means the cotton is prevented from being left behind in the uterus. Professor Sänger prefers a 10 per cent. chloride of zinc solution, or a 30 per cent. argentic nitrate solution, or a solution of iodine. The cauterisation should be repeated every five or six days, if iodine be used, or once a week if zinc chloride or argentic nitrate be used. When, on the other hand, the cervical canal of the uterus is large, he prefers a 50 per cent. solution of chloride of zinc; but in this case the interval between the cauterisations should be 16 to 20 days.

**Perforation from Rapid Dilatation.**—Dr. Auvard[3] ingeniously exonerates the curette from the serious charge of perforation of the uterus, in cases apart from pregnancy or the puerperium; and he insists that it is the rapid dilatation of the cervix which lacerates the uterine wall, and that the curette merely discovers the perforation. He condemns the two-bladed and three-bladed rapid dilators, but expresses trust in Hegar's dilators and in the use of laminaria tents. He says that perforation of the uterus will disappear with the disappearance of the two and three bladed dilators, and not before.

**Backward Displacement of the Uterus.**— Jules Batnaud, adopting Schultze's views as to the causes of uterine displacements, writes on the methods of reducing backward displacements, after the suggestions of Rapin made at the Congress at Rome. He points out[4] that many cases of backward displacement in which this organ is supposed to be adherent to the surrounding viscera are, as a matter of fact, simply held in Douglas' pouch by a combination of atmospheric pressure, the intestines, and the two sacro-uterine ligaments, which imprison it laterally. To dislodge the uterus from this incarcerated position, Rapin advises as follows: On the first two occasions, insert the sound, and rotate in the usual way. If the position remains unchanged the third time, instead of lowering the handle of the sound, press upon the sound from behind forward, and then from below upward, moving the uterus as with a lever. By this manœuvre the fundus is dislodged from the rectum, the *cul de sac* is opened, and the intestines fall into it, and fill it. There is little doubt that many cases regarded as hopelessly adherent might in this way be remedied.

**The Causes of Diseases Peculiar to Women** are apparently surprisingly few in number and limited in character. Ignorance and neglect seem to be the most powerful factors. Dr. C. P. Noble has an interesting article in the August number of the "International Medical Magazine," in which he puts these causes down as five in number. Firstly, the imperfect development of the sexual organs; secondly, gonorrhœa; thirdly, septic inflammation following childbirth; fourthly, lacerations due to childbirth; and, fifthly, such things as constipation, erroneous habits of living, and errors in dress.

In regard to the first of these five causes he lays too much stress apparently on the inadequate development of the organs, due, not to disease, but the simple failure of development, and not enough stress on the arrest of development and the wrong development due to infectious disease during childhood. Scarlet fever, measles, mumps, &c., set up inflammatory processes that often lead to not only a want of development, but to a diseased development of the organs that causes much trouble later on.

Of the second cause, gonorrhœa, recent knowledge has led us all to think greatly of its gravity as a cause, and a frequent cause of disease in the female, even when quite latent. Noeggerath, in 1873, published his paper on "Latent Gonorrhœa in Women," and called attention to its serious ravages. His views met with fierce opposition at the time, but their truth has been established by the work of modern abdominal surgeons.

It is now well known that gonorrhœa is one of the chief causes of uterine, tubal, ovarian, and peritoneal inflammation. The exact percentage has not been estimated on satisfactory data; but our cities give a much higher percentage than rural districts that have a more cleanly living population.

**The Origin of Myomata.**—Kleinwächter[5] points out that the researches of Rösger have thrown light on the development of uterine myomata; he shows that they arise from the muscular coat of the uterine arteries, as might be expected when it is known that, as he pointed out, the embryonic cells of the united Müller's ducts are the common origin of both uterine vessels and the muscle cells of the uterine walls, and that these two develop simultaneously. This tends to elucidate the etiology of those terribly common but mysterious tumours, which are so often the despair of both patient and physician.

**The Cervix During Menstruation.**—Physiological observations of note are made by Dr. Herman, the president of the Obstetrical Society of London, on the change in size of the cervical canal during menstruation. In reading his paper he referred to the different opinions that had been expressed, some saying the canal was made smaller by swelling of its mucous membrane, some that it was made larger by dilatation during menstruation. All these opinions were based mostly on theory, and little on observation. He detailed measurements made when opportunities presented, and came to the following conclusions: (1) That slight spontaneous dilatation of the cervical canal takes place during menstruation; (2) that this dilatation is at its maximum on the third and fourth days of menstruation; (3) that this dilatation takes place in those who menstruate with pain as well as in those who menstruate without, in those who menstruate scantily as well as in those who menstruate copiously, and there is no marked concomitant variation between the amount of dilatation and the amount of pain or the amount of the flow. Dr. Herman regarded menstruation as a miniature labour; as the cervix dilated in labour, so it did in menstruation. These observations speak for themselves, and throw much light on the rational explanation and treatment of dysmenorrhœa.

[1] Intern. Med. Mag., June, 1894. [2] Ibid, July, 1894. [3] Arch. de Tocol. et de Gynæc. [4] Rev. Med. Chi. des Mal. des Femmes. [5] Frauenarzt, Sept., 1894.

